# Hippocampal EUD in primarily irradiated glioblastoma patients

**DOI:** 10.1186/s13014-014-0276-5

**Published:** 2014-12-06

**Authors:** Raphael Bodensohn, Matthias Söhn, Ute Ganswindt, Gabriele Schupp, Silke B Nachbichler, Oliver Schnell, Claus Belka, Maximilian Niyazi

**Affiliations:** Klinik und Poliklinik für Strahlentherapie und Radioonkologie, LMU Klinikum der Universität München, Campus Großhadern, Marchioninistr. 15, D-81377 München, Deutschland; Neurochirurgische Klinik und Poliklinik, LMU Klinikum der Universität München, Campus Großhadern, Marchioninistr. 15, D-81377 München, Deutschland

**Keywords:** Glioblastoma, Hippocampus, Radiation therapy, EUD

## Abstract

**Background:**

Radiation delivery for malignant brain tumors is gradually becoming more precise. Particularly the possibilities of sparing adjacent normal structures such as the hippocampus are increasing. To determine its radiation exposure more exactly, the equivalent uniform dose (EUD) of the hippocampus was compared with further treatment parameters. This way sparing options could be found.

**Methods:**

From the database of the University hospital of Munich 61 glioblastoma patients were selected who received primary radiotherapy in 2011. General data about the etiology, treatment course, survival of the patients and dose parameters were retrieved.

**Results:**

In a linear regression analysis the side of the tumor (left hippocampus: p < 0.001/right hippocampus: p = 0.009) and its temporal location (left hippocampus: p = 0.015/right hippocampus: p = 0.033) were identified as factors with a significant influence on the EUD of the respective hippocampus. Besides this, the size of the planning target volume (PTV) and the EUD of the hippocampus correlated significantly (p = 0.027; Pearson correlation = 0.291). The median PTV size of the tumor in the right hemisphere was 386.1 ml (range 131.2–910.7 ml), and in the left hemisphere 291.3 ml (range 146.0–588.9 ml) (Kruskal–Wallis test: p = 0.048). A dose quartile analysis showed that 31 patients had a high dose exposure of the hippocampus on one side while having a moderate dose exposure in the other side.

**Conclusions:**

The radiation exposure of the respective hippocampus is dependent on the side where the tumor is located as well as on whether it is temporally located. The exposure of the contralateral hippocampus is further dependent on multiple additional factors – nevertheless a reasonable protection seems to be possible in about half of all cases.

## Background

In spite of therapeutic advancements, glioblastoma multiforme (GBM) remains the most aggressive primary brain tumor with dismal prognosis [1-4]. Nevertheless, the probability of survival has improved in recent years which could epidemiologically be retraced to the introduction of temozolomide and partly bevacizumab. These chemotherapeutic agents have shown to be useful as salvage therapy and e.g. in combination to re-irradiation [5-7] to the therapy of GBM as statistically shown by Wachtel and Yang [8].

The current standard treatment of GBM consists of a multimodal approach consisting of surgical resection (if feasible), radiotherapy and chemotherapy with temozolomide [1,9]. However, a significant prognostic advantage is only seen if patients undergo a gross total resection (GTR); partial resections have no proven advantage over a biopsy alone [10]. The O^6^-Methylguanine-DNA Methyltransferase (MGMT) methylation status has been shown to be of prognostic relevance as it seems to enhance the efficacy of temozolomide [2,11]. There is however still doubt if the MGMT status should be used routinely as a prognostic marker, due to the lack of studies in this matter [12]. In elderly patients, however, concomitant temozolomide should be used more cautiously [13]. Fariselli et al. suggest a hypofractionated regimen with 45 Gy as a safe option for elderly patients [14]. However the Nordic trial and NOA-08 suggest additionally a monotherapy with temozolomide for elderly patients with a methylated MGMT promoter sequence [15,16]. Due to more accurate radiation delivery, normal brain structures can be spared to a greater extent. Next to routinely spared organs-at-risk (OARs) such as the brainstem, the optic chiasm, the inner ear or the eye lenses, recent studies dealt with the identification and sparing of other nerval structures like, for instance, the hippocampus [17-20]. As hippocampal neurogenesis seems to play an important role in cognition, an alteration of the proliferation of neural cells could be a reason for cognitive impairment as studies both on mice and on humans have suggested [21,22]. As a cognitive loss is, together with depression and anxiety, an important reason for a decrease in quality of life, a functioning hippocampus is important for the patient’s personal situation [23,24]. Thus, successfully sparing this structure could be of great advantage not only for patients with GBM, but also for patients with other brain tumors with more favorable prognosis where long-term side effects may be of higher relevance. Modern radiation technology such as IMRT (intensity modulated radiotherapy) seems to offer a good way for an improved sparing [25].

This study retrospectively assesses DVH (doses volume histogram) parameters after primary radiotherapy of GBM patients with an emphasis on the hippocampus. In order to determine possible reasons for high exposure of the hippocampus different dose/volume parameters of the hippocampus were compared with those of the brain and the PTV, as well as other parameters.

## Methods

The cohort consists of 61 GBM patients who were treated for the first time with radio(−chemo-) therapy at the University hospital of Munich, Department of Radiation Oncology in 2011. All patients had a histologically and radiologically (using magnetic resonance imaging (MRI) with contrast media) proven GBM and had a treatment plan based on Oncentra® (by Elekta, Stockholm, Sweden). The contours of the patients’ hippocampi were outlined directly in the Oncentra® cases in order to retrospectively determine its radiation exposure. Some patients’ hippocampi could not be located in their entirety due to the size and location of the tumor. For these patients, the hippocampus on the tumor’s side was left out partly or entirely, thus only leaving the contralateral side to be contoured. The statistical data were analyzed with IBM SPSS Statistics® Version 21.

### Contouring of the hippocampus

In order to outline the hippocampus contours systematically, the guide by Chera et al. was used [26]. It is suggested to use the T1 sequence of the MRI and look for a slice in the transverse plane which shows the temporal horn of the lateral ventricle. Here, the caudal part of the hippocampus can be seen as the hypointense structure next to the amygdala. From there, one can follow the hippocampus towards cranial alongside the ventricle. In this study, however, it seemed to be easier to start contouring the hippocampus from the most cranial part as in many cases the caudal part was consumed by the tumor or its edema. Near the fornix a thin part of gray matter can be found, which then follows the lateral ventricle until the amygdala is caudally reached.

### Radiotherapy

Radiotherapy consisted of a conventionally fractionated regimen using 3D conformal radiotherapy (3D-CRT), with the delivery of a total dose of 60 Gy in 6 weeks, in a once-daily schedule of 2 Gy per fraction for a total of 30 fractions. Patients were treated using megavoltage equipment, such as linear accelerator beams with nominal energy of 6MV. All patients were immobilized in supine position using a commercially available thermoplastic mask system. Computed imaging (CT) data, reconstructed in 2.5 mm or 3 mm slice thicknesses, were coregistered with available MR images in T2 or fluid attenuation inversion recovery and T1 post-contrast weighting. The gross tumor volume (GTV) consisted of the entire visible tumor at preoperative contrast-enhanced MRI, the clinical target volume (CTV) included the entire enhanced tumor (according to preoperative contrast CT or MRI) plus a 20 mm margin including the perifocal edema (5 mm PTV margin). Treatment planning and dose calculation were based on reports 50 and 62 of the International Commission on Radiation Units and Measurements.

### Equivalent uniform dose concept

In order to compare two DVHs and their associated effects more differentially, the equivalent uniform dose (EUD) concept has been introduced by Niemierko [27]. The EUD is the radiation dose which has, for an idealized homogeneous dose distribution, the same clinical effect as it is accomplished by a corresponding heterogeneous dose distribution. Former concepts only considered absolutely or piecewise homogeneous dose distributions, which, however, can rarely be achieved in vivo [28]. By taking the heterogeneous dose distribution into account, the EUD can depict the activity in irradiated tissues in a more realistic way [29]. Thus, using the EUD, the clinical outcome of avoidance structures can be estimated more accurately. For instance, the EUD has already been used to determine certain treatment parameters of malignant glioma [30].

In a generalized form, which describes the tumor cells more specifically, the following formula is used [31]:

$$ EUD={\left({\displaystyle \sum_j{v}_j{D}_j^k}\right)}^{\frac{1}{k}} $$

where the sum is calculated over all dose bins (υ_j_,D_j_) of the differential DVH, and k is the volume-effect parameter (range, k∊ [1… ∞]). According to this formula, in case of k → ∞ the EUD is the maximum dose (no volume effect), and in case of k = 1 the EUD is the mean dose (large volume effect) [31]. In this study following parameters for k were used: for the brain k = 5 and for all the other structures k = 12 [31].

## Results

### Patient characteristics

Altogether, 61 patients were identified from the department’s database, whereby 36 were male (59.0%) and 25 female (41.0%). 27 patients were aged 60 years or below (44.3%), and 34 patients were older than 60 years (55.7%). Five patients (8.2%) had a bilateral tumor, 29 (47.5%) a left-sided, and 27 a right-sided tumor (44.3%). The exact locations can be found in Table 1. 46 patients (75.4%) received a chemotherapy with temozolomide. 22 patients (36.1%) received a gross total resection, two patients a subtotal resection (3.3%), and 37 patients (60.7%) a biopsy only. More details can be found in Table 2.

**Table 1:** **Distribution of the tumor location**

**Tumor location**	**Left**	**Right**	**Bilateral**	**Total**
**Frontal**	7	8	4	19
**Temporal**	11	9	0	20
**Parietal**	5	2	0	7
**Occipital**	0	2	1	3
**Central**	3	3	0	6
**Frontotemporal**	0	1	0	1
**Frontoparietal**	1	0	0	1
**Temporoparietal**	1	0	0	1
**Temporoocipital**	1	1	0	2
**Parietooccipital**	0	1	0	1
**Total**	29	27	5	61

**Table 2:** **Patient characteristics**

		**Frequency**	**Percentage**
**Sex**	Male	36	59.0
	Female	25	41.0
**Kind of operation**	GTR	22	36.1
	PE	37	60.7
	STR	2	3.3
**Temozolomide**	Yes	46	75.4
	No	15	24.6
**Karnofsky index**	50	3	4.9
	60	4	6.6
	70	20	32.8
	80	11	18.0
	90	12	19.7
	100	11	18.0
**MGMT promoter methylated**	Yes	21	34.4
	No	31	50.8
	Partially	9	14.8
**IDH1/2 mutated**	No data	5	8.2
	Yes	4	6.6
	No	52	85.2
**LOH 1p/19q allelic**	No data	10	16.4
	Yes	2	3.3
	Only 19q	4	6.6
	Only 1p	3	4.9
	No	42	68.9
**Interuption of temozolomide**	No data	19	31.1
	Yes	13	21.3
	No	29	47.5
**Age**	= < 60 y	27	44.3
	> 60 y	34	55.7

### Overall survival

**Figure 1:**
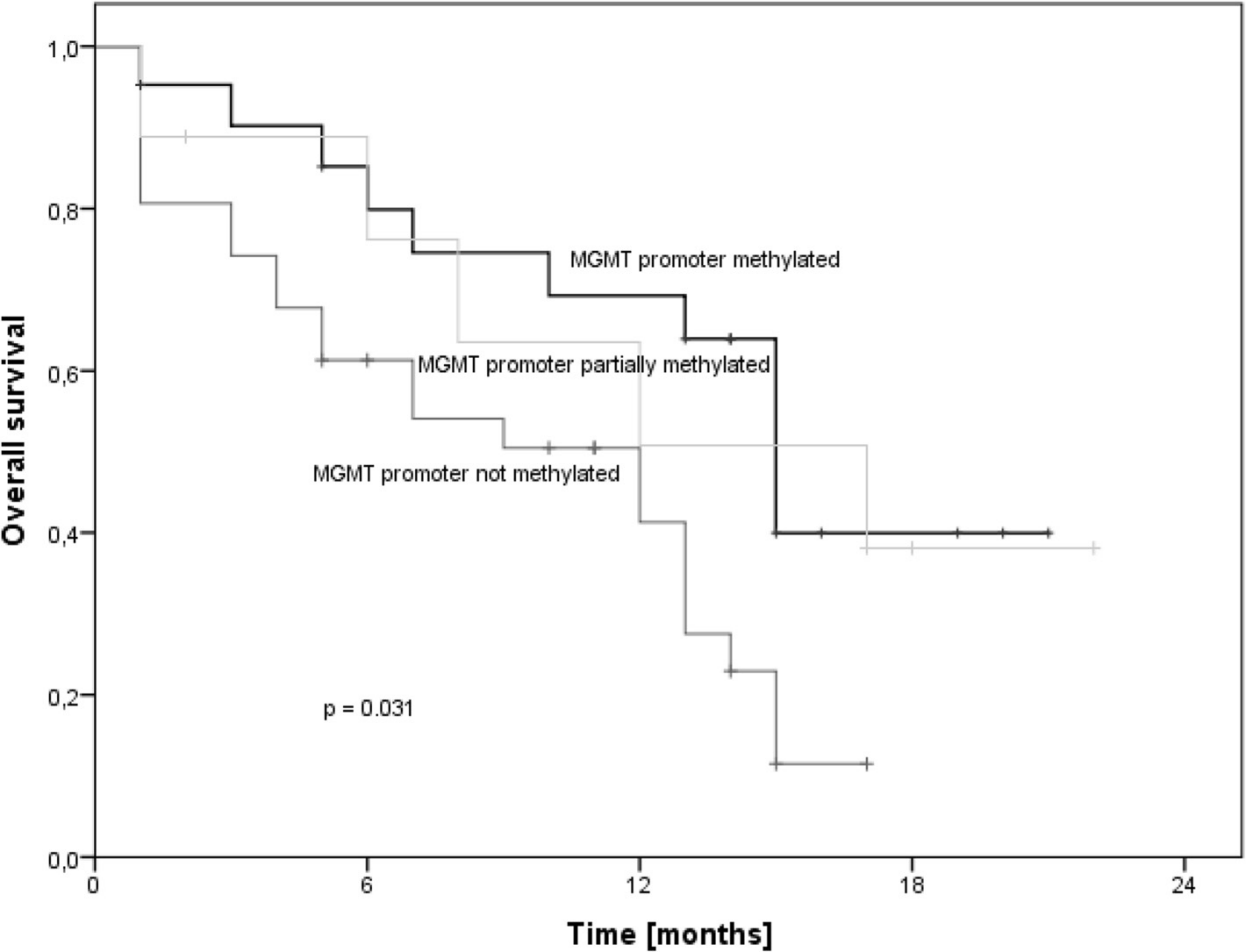
**Survival depending on MGMT promotor methylation.**

**Figure 2:**
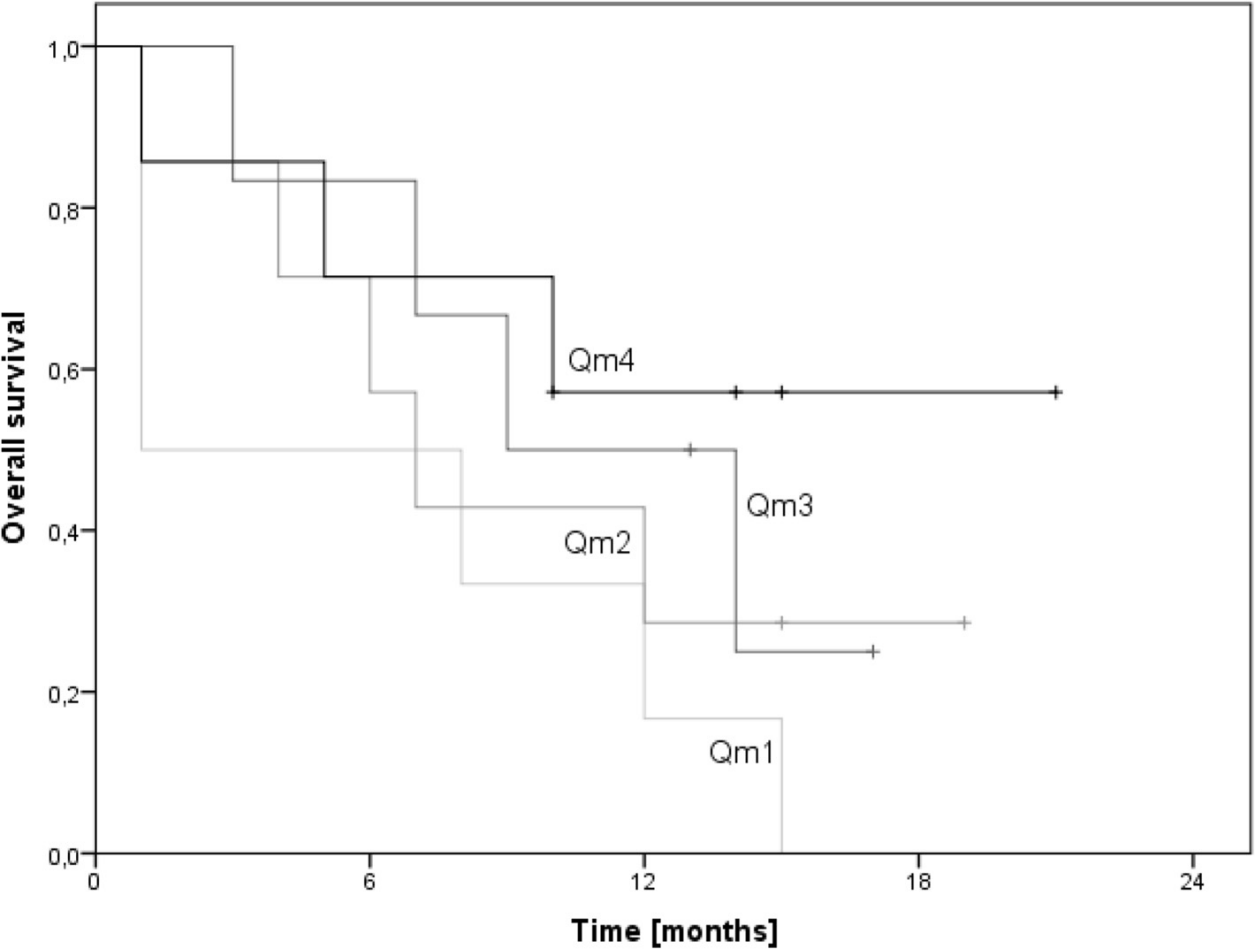
**Survival depending on the maximum dose to the right hippocampus for patients with a right sided tumor.** Q_m_1 (0–42.3 Gy); Q_m_2 (42.3–58.7 Gy); Q_m_3 (58.7–59.9 Gy); Q_m_4 (>59.9 Gy).

The median overall survival rate of the cohort was 13.0 months (95% confidence interval: 10.2–15.8). For patients aged 60 years or below the estimated median survival was 15.0 months (95% confidence interval: 13.0–17.0); for patients above 60 years of age the median survival rate was 8.0 months (95% confidence interval: 4.2–11.8) (log-rank: p = 0.052). Male patients had a median survival rate of 13.0 months (95% confidence interval: 9.4–16.6), identical to that of female patients (95% confidence interval: 10.0–16.0) (log-rank: p = 0.893). Patients treated with temozolomide had a median survival rate of 13.0 months (95% confidence interval: 11.2–14.8) compared to patients not treated with temozolomide who had a median survival rate of 8.0 months (95% confidence interval: 1.0–15.0) (log-rank: p = 0.133). The size of the tumor had no influence on the patients’ survival (log-rank: p = 0.893). The patients whose MGMT promoter sequence was methylated had a median survival rate of 15.0 months (95% confidence interval: 12.9–17.1) and a mean survival rate of 14.4 months (95% confidence interval: 11.3–17.4), the patients with an only partially methylated MGMT promoter sequence had a median survival rate of 17.0 months (95% confidence interval: 5.1–29.0) and a mean survival rate of 14.0 months (95% confidence interval: 8.8–19.1), whereas the patients whose MGMT promoter sequence was not methylated had a median survival rate of 12.0 months (95% confidence interval: 5.5–18.5) and a mean survival rate of 9.0 (95% confidence interval: 6.9–11.1) (log-rank: p = 0.031) (Figure 1). Moreover the cox regression analysis (p = 0.029) showed a slight but significant indication that the survival rate is dependent on the maximum dose supplied to the ipsilateral hippocampus for patients with a right sided tumor. This could however not be shown for the rest of the cohort. The Kaplan-Meier plots did not show overall significant results in this matter. Nevertheless the survival of the patients with a right sided tumor and a higher dose (Q_m_4) to the ipsilateral hippocampus showed a trend towards increased survival compared to patients with a low dose (Q_m_1) (log-rank: p = 0.076) (Figure 2).

### Treatment parameters

The patients’ median planning target volume (PTV) size was 358.1 ml. The median cerebral volume (N = 60) was 1351.6 ml. Furthermore, the mean dose of the brain was median 33.2 Gy with a median maximum dose of 63.1 Gy, and a median EUD of 46.9 Gy. The median hippocampus total volume (N = 58) was 2.5 ml. The exact distribution of the hippocampal dose values can be found in Table 3.

**Table 3:** **Treatment parameters**

		**N**	**Average**	**Median**	**Standard deviation**	**Minimum**	**Maximum**
**PTV [ml]**		61	351.3	358.1	142.4	131.2	910.7
**Brain**	Volume [ml]	60	1350.2	1351.6	143.4	1033.1	1766.8
	Mean dose [Gy]	60	31.6	33.2	8.0	15.5	45.5
	Maximum dose [Gy]	60	61.2	63.1	5.8	40.7	65.3
	V45 [Gy]	60	33.2	33.1	15.1	0.0	61.0
	V50 [Gy]	60	29.0	29.5	14.4	0.0	56.8
	V60 [Gy]	60	10.3	10.8	6.2	0.0	24.9
	EUD [Gy]	59	46.1	46.9	5.1	31.0	52.5
**Hippocampus total**	Volume [ml]	58	2.7	2.5	0.8	1.5	4.7
	Mean dose [Gy]	58	33.2	38.5	15.1	1.9	53.5
	Maximum dose [Gy]	58	51.3	58.9	16.2	2.7	61.1
	EUD [Gy]	58	46.1	53.2	14.7	2.1	57.8
**Hippocampus left**	Mean dose [Gy]	59	37.4	41.6	19.6	1.7	60.1
	Maximum dose [Gy]	59	43.8	52.5	19.0	2.2	61.1
	EUD [Gy]	59	40.9	48.0	18.3	1.8	59.9
**Hippocampus right**	Mean dose [Gy]	59	34.0	33.4	18.7	2.1	64.8
	Maximum dose [Gy]	59	40.9	44.6	18.6	2.7	61.5
	EUD [Gy]	59	37.6	38.4	17.4	2.3	61.2

### Correlation of dose parameters

**Figure 3:**
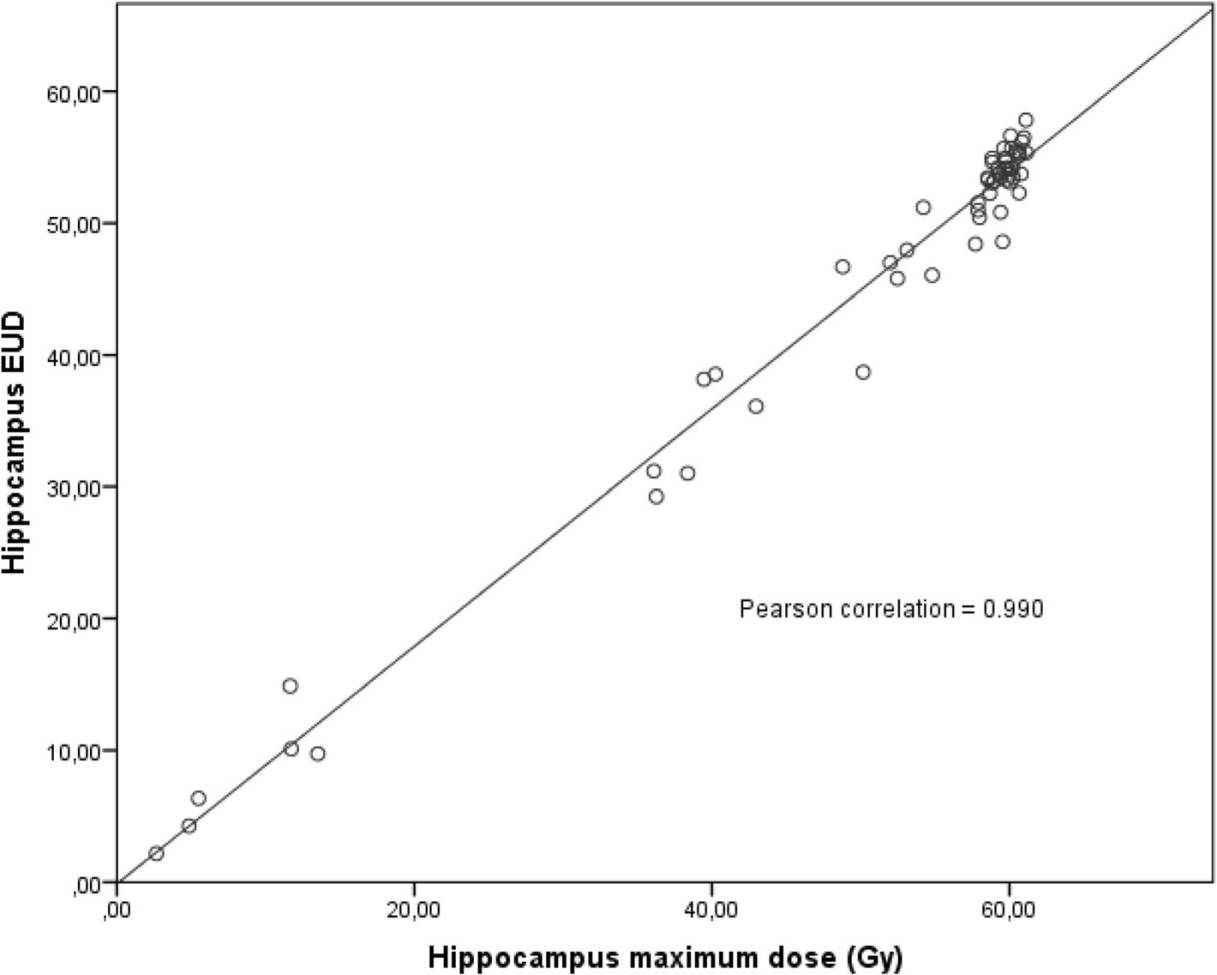
**Correlation between hippocampus EUD and hippocampus maximum dose.**

There was a significant correlation between PTV size and total hippocampus EUD (N = 58; Pearson correlation = 0.291; p = 0.027). Additionally, there was a significant correlation between the PTV size and the mean dose of the brain (N = 60; Pearson-correlation = 0.742; p < 0,001). The maximum dose to which the brain was exposed to correlated significantly with the V60 dose volume of the brain (N = 60; Pearson correlation = 0.645; p < 0.001). Furthermore, the brain EUD significantly correlated with the mean dose of the brain (N = 59; Pearson correlation = 0.778; p = 0.001). As expected the brain EUD was significantly correlated with the maximum dose of the brain (N = 59; Pearson correlation = 0.775; p < 0.001). Moreover, the hippocampus EUD correlated significantly with the mean dose of the hippocampus (N = 58; Pearson correlation = 0.752; p < 0.001). The bilateral hippocampus EUD was also significantly correlated with the maximum dose of the hippocampus (N = 58; Pearson correlation = 0.990; p < 0.001) (Figure 3). Additionally, the left hippocampus EUD was significantly correlated with the maximum dose of the left hippocampus (N = 59; Pearson correlation = 0.991; p < 0.001). The left hippocampus EUD was also correlated with the mean dose of the left hippocampus (N = 59; Pearson correlation = 0.974; p < 0.001). For the right side similar results were obtained: The right hippocampus EUD was significantly correlated with the mean dose of the right hippocampus (N = 59; Pearson correlation = 0.946; p < 0.001). A significant correlation also was found between the right hippocampus EUD and the maximum dose of the right hippocampus (N = 59; Pearson correlation = 0.975; p < 0.001). Apart from that, the brain EUD was significantly correlated with the hippocampus EUD (N = 59; Pearson correlation = 0.399; p = 0.002). The results of the regression analysis showed a significant influence of the side of the tumor (left: p < 0.001; right: p = 0.009) and the temporal location of the tumor vs. another location (left: p = 0.045; right: p = 0.033) on the EUD of the respective hippocampus. The median volume of the tumors on the right side was 386.1 ml (range 131.2–910.7 ml), the median of the left side was 291.3 ml (range 146.0–588.9 ml) (Kruskal Wallis test: p = 0.048).

### Dose-quartile analysis of the hippocampus

In order to determine which patients had a bilateral high dose exposition, the EUD of each hippocampus was divided into quartiles (Table 4). The patients were separated in patients with left, right or bilateral tumor location. Then it was assessed how many patients had a certain quartile on one side while having a certain quartile on the other side (for example there were 11 patients with a left sided tumor which had Q3 + 4 on the left hippocampus and at the same time Q2 on the right hippocampus). The most important message of this analysis was that there were in total 31 patients who had a high dose exposition (Q3 + 4) on one side but at the same time a moderately to high exposition on the other side (Q2 and Q3 + 4). The EUD of the contralateral hippocampi of these patients could be reduced below the cutoff value of Q1 (right side: 25.7 Gy; left side: 27.5 Gy) which was regarded as low-dose exposition. The other half of the patients either already had an EUD below the cutoff value of Q1 on the contralateral hippocampus or have a bilateral tumor making it impossible to spare one side.

**Table 4:** **Quartiles of the hippocampus EUD**

**Side of the tumor**			**Quartiles right hippocampus**			**Total**
			**Q1**	**Q2**	**Q3 + 4**	
**Both sides**	**Quartiles left hippocampus**	Q2	0	1	2	3
		Q3 + 4	0	0	2	2
	**Total**		0	1	4	5
**Left**	**Quartiles left hippocampus**	Q1	3	0	0	3
		Q2	1	0	0	1
		Q3 + 4	7	11	5	23
	**Total**		11	11	5	27
**Right**	**Quartiles left hippocampus**	Q1	3	2	6	11
		Q2	0	0	10	10
		Q3 + 4	0	1	4	5
	**Total**		3	3	20	26
**Total**	**Quartiles left hippocampus**	Q1	6	2	6	14
		Q2	1	1	12	14
		Q3 + 4	7	12	11	30
	**Total**		14	15	29	58

Right: Q1 (0–25.7 Gy), Q2 (25.7–38.4 Gy), Q3 + 4 (>38.4 Gy); Left: Q1 (0–27.5 Gy), Q2 (27.5–47.9 Gy), Q3 + 4 (>47.9 Gy).

## Discussion

If one attempts to achieve a reasonable sparing of the hippocampus, it is reasonable to know in which constellations this is actually possible without risking missing tumor cells during radiation and thus deteriorating patient’s outcome. In order to determine the possibilities of sparing the hippocampus, it is important to know relevant parameters influencing its radiation exposure. Due to its anatomy it would be logical to assume that the hippocampus is more strongly exposed to radiation if the tumor is in temporal position and that the ipsilateral hippocampus is more strongly affected by radiation, which has both been confirmed by the results of this study. Thus, presumably hippocampal sparing can best be achieved with non-temporal tumors and contralateral localization.

As already mentioned within the introduction, recent studies suggest a connection between the cognitive function of the hippocampus and local neurogenesis, which possibly is the reason for the sensitivity of this structure to radiation [21,22]. Concerning a functional asymmetry of the hippocampus, research has recently been done: the dominance of the left hippocampus in verbal memory has been shown in many studies; the dominance in figural and spacial memory of the right hippocampus has often been suggested but has yet hardly been proven [32]. The exact function of the hippocampus and its reaction to radiation is a rather recent topic. Thus, the question of sparing this structure has also only been discussed recently.

G. T. Armstrong et al. showed that patients who were irradiated in their childhood due to brain cancer had more neurocognitive impairments if they were irradiated in the temporal lobe [33]. The impairments, however, only seem to be relevant when irradiating with high doses as a study by E. Olsson et al. showed no correlation of hippocampal exposure and quality of life for patients who received low dose irradiation [34]. If a sparing has to be achieved, a reduction of the margin could be a good method in order to lower the dosage the hippocampus is exposed to [35]. M. B. Pinkham et al. suggest in a 2014 study with WHO grade II and III gliomas that one should use IMRT in order to spare the hippocampus as effectively as possible [36]. The sparing, however, does not seem to be only beneficial for the patient: the subgranular zone of the hippocampus contains stem cell niches which could be a reason for recurrences of high grade tumors as P. Evers et al. have shown [37]. Recent studies even showed an improvement of the overall survival, if the ipsilateral subventricular zone (including the stem cell niches of the subgranular zone of the hippocampus) was irradiated with a higher dose [38,39]. Our data also have shown a similar slight tendency only regarding the dose applied to the right hippocampus. I. Gibbs et al. however express doubt if this information really should be used for therapy because of the neurocognitive side effects and the possibility of this being a “trojan horse” [40]. But looking at the information at hand, it seems to be best for high grade tumors to spare the contralateral hippocampus as well as possible in order to reduce the cognitive impairment but to include the ipsilateral hippocampus due to the risk of recurrences. So in total a sparing could be discussed in about half of all cases. For low grade tumors with a negligible risk for recurrence, a sparing of both sides of the hippocampus, using reduction of margin [35] and/or IMRT [36], should be considered.

To evaluate the true gain of hippocampal sparing, more prospective studies would still be needed. Especially the effect of radiation on the patients’ cognition compared to dose-volume parameters still needs to be evaluated. For that reason, our institution is at present working on a study to assess this issue properly. Advanced knowledge about cognitive effects of cerebral radiation and a resulting gain in precision could reduce the long-term side effects for patients and thus make the radiation therapy a less strenuous treatment.

A surprising result was that the tumor’s size seemed to depend on the side on which the tumor was located: tumors were significantly larger if they were localized in the right hemisphere. It would be interesting to see if this phenomenon can be reproduced in a larger cohort.

## Conclusions

Looking at the results the temporal location and the side of the tumor seem to be the greatest influence on the radiation exposure of the hippocampus. A possible therapy option could be to spare the contralateral hippocampus, in order to maintain the patients’ memory function as good as possible, but to include the ipsilateral hippocampus, due to the risk of recurrences through the local stem cell niches. The study shows that a sparing would at least make sense in about half of all cases, while the other half either cannot be spared due to a bilateral tumor, or has no need to be spared due to a rather low dose to the contralateral hippocampus.

### Consent

The scientific usage of retrospective data has been explicitly allowed by the Bavarian federal law. Additionally all patients agreed that their scientific data could be used. No experimental research on humans or animals has been performed or reported.

The declaration of Helsinki has been obeyed in all points.
